# Long-term efficacy of mepolizumab in patients with eosinophilic granulomatosis with polyangiitis: a propensity score matching analysis in the multicenter REVEAL cohort study

**DOI:** 10.3389/fimmu.2024.1457202

**Published:** 2024-10-02

**Authors:** Mayu Shiomi, Ryu Watanabe, Shogo Matsuda, Takuya Kotani, Ayana Okazaki, Yuichi Masuda, Tsuneyasu Yoshida, Mikihito Shoji, Ryosuke Tsuge, Keiichiro Kadoba, Ryosuke Hiwa, Wataru Yamamoto, Akitoshi Takeda, Yoshiaki Itoh, Motomu Hashimoto

**Affiliations:** ^1^ Department of Neurology, Osaka Metropolitan University Graduate School of Medicine, Osaka, Japan; ^2^ Department of Clinical Immunology, Osaka Metropolitan University Graduate School of Medicine, Osaka, Japan; ^3^ Department of Internal Medicine (IV), Osaka Medical and Pharmaceutical University, Takatsuki, Japan; ^4^ Department of Rheumatology and Clinical Immunology, Graduate School of Medicine, Kyoto University, Kyoto, Japan; ^5^ Department of Health Information Management, Kurashiki Sweet Hospital, Kurashiki, Japan

**Keywords:** eosinophilic granulomatosis with polyangiitis, glucocorticoid-sparing, mepolizumab, multicenter cohort, survival rate

## Abstract

**Background:**

Mepolizumab (MPZ) has demonstrated efficacy in clinical trials for eosinophilic granulomatosis with polyangiitis (EGPA); however, few studies compare the disease course between patients treated with MPZ (MPZ group) and those who were not treated with MPZ (non-MPZ group) in real-world settings.

**Objectives:**

This study aimed to compare the disease course and outcomes between the two groups and assess the long-term efficacy of MPZ in a multicenter cohort in Japan. Methods: We enrolled 113 EGPA patients registered in the cohort until June 2023. Data on clinical characteristics, disease activity, organ damage, treatments, and outcomes were retrospectively collected. To minimize potential confounding factors, we conducted propensity score matching (PSM).

**Results:**

After PSM, 37 pairs of matched patients were identified. Clinical characteristics, including age at disease onset, sex, disease duration at last observation, antineutrophil cytoplasmic antibody positivity at disease onset, Birmingham Vasculitis Activity Score (BVAS) at disease onset, and Five-factor score at disease onset, were comparable between the groups. The median BVAS at the last observation was 0 in both groups; however, more cases in the non-MPZ group exhibited elevated BVAS, resulting in a significantly higher BVAS in the non-MPZ group at the last observation (median; MPZ group: 0, non-MPZ group: 0, p=0.028). The MPZ group had significantly lower glucocorticoid (GC) doses at the last observation (median; MPZ group: 4 mg/day, non-MPZ group: 5 mg/day, p=0.011), with a higher proportion achieving a GC dose ≤ 4 mg/day at the last observation (MPZ group: 51.4%, non-MPZ group: 24.2%, p=0.027). Three models of multivariable logistic regression analyses were performed to identify factors associated with GC doses ≤ 4 mg/day at the last observation. In all models, achieving a GC dose ≤ 4 mg/day was positively associated with MPZ administration and inversely associated with asthma at disease onset. Finally, we evaluated the survival rates between the groups, and the 5-year survival rates were significantly higher in the MPZ group compared to the non-MPZ group (MPZ group: 100%, non-MPZ group: 81.3%, p=0.012).

**Conclusion:**

Mepolizumab not only contributes to disease activity control but also reduces the GC dose, which may lead to improved survival in EGPA patients.

## 1 Introduction

Eosinophilic granulomatosis with polyangiitis (EGPA), first reported in 1951, is currently recognized as a small-to-medium-sized necrotizing vasculitis, classified as one of the antineutrophil cytoplasmic antibody (ANCA)-associated vasculitis (1, 2). Myeloperoxidase (MPO)-ANCA is detected in 30–35% of patients. It is histologically characterized by extravascular granulomas and eosinophilia in both blood and tissues, which can lead to systemic organ damage, including asthma, peripheral neuropathy, cardiomyopathy, gastroenteritis, and glomerulonephritis (3–5). Glucocorticoid (GC) has formed the cornerstone of treatment for EGPA. In cases of severe disease, immunosuppressive agents (IS) such as cyclophosphamide or alternatively rituximab are added to high-dose GC (6–8). Although disease control can be achieved, a non-negligible proportion of patients still experience relapses upon tapering or discontinuation of these medications. Furthermore, long-term use of these agents imposes substantial burdens on patients due to adverse events, such as infections and secondary malignancies.

In recent years, several therapeutic agents targeting interleukin (IL)-5 have been developed. IL-5 is essential for eosinophil differentiation, maturation, and extravasation (5, 9, 10). Mepolizumab (MPZ) is a humanized monoclonal antibody against IL-5 that inhibits the interaction between IL-5 and its receptor on the surface of eosinophils (4). The MIRRA trial, a randomized controlled trial conducted in 2017, demonstrated that, compared to placebo, MPZ 300 mg every 4 weeks showed a higher remission induction rate and GC-sparing effect for relapsing and treatment-refractory patients (11). Based on this trial, MPZ is currently recommended for remission induction for relapsing and treatment-refractory patients without organ- or life-threatening manifestations as well as for remission maintenance after remission induction (7, 8).

However, the clinical characteristics of the patients enrolled in the MIRRA trial differ from real-world clinical scenarios in several aspects. Particularly, the ANCA positivity was as low as 19% at disease onset, and patients with organ- or life-threatening manifestations were excluded from the trial (11). Therefore, there is an urgent need to evaluate the effectiveness of MPZ in real-world clinical settings. In addition, the observation period of the MIRRA trial was relatively short (52 weeks). Thus, the long-term effectiveness of MPZ, by comparing the disease course of MPZ-treated and untreated patients, should be evaluated in real-world settings.

In this study, patients were divided into two groups: the MPZ group, who were introduced to MPZ during the disease course, and the non-MPZ group, who were not introduced to MPZ. To match the patients’ backgrounds, propensity score matching was performed. Disease activity, GC doses, recurrence rates, and survival rates between the two groups were compared in a real-world patient cohort in Japan.

## 2 Materials and methods

### 2.1 Patients

This retrospective multicenter study was conducted using the Registry of Vasculitis Patients to Establish the REAL-WORLD Evidence (REVEAL) cohort (12–14). The REVEAL cohort is an observational registry comprising patients with vasculitis from the Kansai region of Japan. The dataset consists of information from three participating institutions: Osaka Metropolitan University, Kyoto University, and Osaka Medical and Pharmaceutical University. A total of 113 EGPA patients who had a history of medical visits to the relevant facilities by June 2023 were registered based on meeting at least one of the following three criteria: Lanham criteria (1984) (15), American College of Rheumatology (ACR) classification criteria (1990) (16), and ACR and the European Alliance of Associations for Rheumatology (EULAR) criteria (2022) (17). Based on shared decision-making between attending physicians and patients, 60 patients received MPZ during remission induction, remission maintenance, or relapse phase. Fifty-eight patients received subcutaneous injections of 300 mg every 4 weeks, while one patient received 100 mg, and another received 200 mg at the discretion of their attending physician.

This study was conducted in accordance with the principles outlined in the Helsinki Declaration. Ethical approval was obtained from the Ethics Committees of Osaka Metropolitan University (Approval No. 2023-027) and the participating institutions, Kyoto University (Approval No. R1540) and Osaka Medical and Pharmaceutical University (Approval No. 1529). Informed consent was obtained from all participants except for those who visited Osaka Metropolitan University Hospital between 2008 and 2021 because the Ethics Committee of Osaka Metropolitan University waived the requirement for informed consent due to the anonymous nature of the data.

### 2.2 Clinical evaluation

The clinical data were retrospectively extracted from medical records, including demographic information such as age at disease onset, sex, absolute eosinophil count, MPO-ANCA status, and GC dose, as well as laboratory test results and treatment. The 2009 five-factor score (FFS) (18), the Birmingham Vasculitis Activity Score (BVAS) version 3 (19), and the vasculitis damage index (VDI) (20) were used for prognostic evaluation, disease activity, and irreversible organ damage, respectively. In this study, relapse was defined as a worsening of EGPA diagnosed by the attending physician, requiring an increase in GC dose and/or the addition of IS. The relapse symptoms included exacerbation of vasculitis, asthma, and sinusitis. The relapse manifestations were categorized into two types: recurrences of systemic vasculitis and isolated exacerbations of asthma or otolaryngological symptoms (8).

### 2.3 Statistical analysis

Propensity score matching (PSM) is a popular method applied in clinical research to reduce selection bias by adjusting for potential confounding factors (21, 22). PSM was conducted employing a multivariable logistic regression model to reduce selection bias and match the patients’ backgrounds, using the following key variables: age, sex, disease duration, FFS score, BVAS, absolute eosinophil count, and C-reactive protein (CRP) at disease onset. The patients were divided into two groups based on their propensity scores.

The data were presented using medians and interquartile ranges (IQR) for continuous variables and numbers and percentages (%) for categorical variables. Fisher’s exact test or the Mann-Whitney U test was used to compare differences between groups. A p value <0.05 in a two-sided test was considered statistically significant. In the multivariable analysis, statistical significance was determined by the Bonferroni correction to adjust for multiple testing (23). The Kaplan-Meier method was used to examine survival and relapse-free survival rates, and log-rank tests were performed to detect the significance of differences between groups. Using multivariable logistic regression analysis, odds ratios and 95% confidence intervals for variables associated with GC dose ≤ 4 mg/day were calculated. All statistical analyses were performed using EZR (Saitama Medical Centre, Jichi Medical University, Saitama, Japan) (24) and GraphPad Prism 10 (GraphPad Software, La Jolla, CA, USA).

## 3 Results

### 3.1 Patient background before PSM

Of the 113 patients registered in our database, sixty patients were administered MPZ during the disease course. Excluding one patient with an unknown start date of MPZ, MPZ was introduced at a median of 32 months after the onset of the disease. **Table 1** summarizes the background of the patients in the MPZ (n=60) and non-MPZ (n=53) groups. Before matching, the MPZ group exhibited a significantly younger age at onset compared to the non-MPZ group; however, other baseline characteristics were comparable between the two groups (**Table 1**).

**Table 1:** Baseline characteristics of participants before and after propensity score matching.

Variable	before PSM			after PSM		
	MPZ (n = 60)	non-MPZ (n = 53)	p	MPZ (n = 37)	non-MPZ (n = 37)	p
First case/recurrence case, n (%) ^a)^	48/11 (80.0)/(18.3)	46/7 (86.8)/(13.2)	0.46	32/5 (86.5)/(13.5)	32/5 (86.5)/(13.5)	1.00
Age at onset (year), median (IQR)	54.5 (43.5, 65.3)	61.0 (50.0, 73.0)	0.014*	63.0 (51.0, 67.0)	59.0 (48.0, 71.0)	0.68
Female, n (%)	33 (55.0)	28 (52.8)	0.85	21 (56.8)	16 (43.2)	0.35
Disease duration at the last observation (months), median (IQR)	72.0 (36.0, 99.0)	89.0 (48.0, 116.0)	0.29	72.0 (48.0, 108.0)	92.0 (35.0, 132.0)	0.51
FFS at onset, median (IQR)	1 (0, 1)	1 (0, 2)	0.58	1 (1, 2)	1 (0, 2)	0.30
BVAS at onset, median (IQR)	16.0 (11.0, 20.5)	16.0 (11.0, 21.0)	1.00	17 (13, 21)	15 (9, 20)	0.16
White blood cell at onset (/μl), median (IQR)	15370 (9050, 21740)	13600 (10500, 19270)	0.67	15740 (12500, 21480)	13170 (10500, 18960)	0.25
Eosinophil at onset (/μl), median (IQR)	4969 (1511, 11681)	3714 (1399, 9625)	0.77	7125 (2100, 11764)	4200 (1302, 9239)	0.43
Hb at onset (g/dL), median (IQR)	12.8 (12.0, 14.1)	12.7 (11.7, 14.0)	0.53	12.6 (11.7, 13.2)	12.9 (11.8, 14.1)	0.29
eGFR at onset (mL/min/1.73), median (IQR)	83.8 (66.9, 101.5)	80.7 (64.3, 95.4)	0.46	81.5 (59.4, 97.7)	89.7 (69.9, 99.2)	0.36
CRP at onset (mg/dl), median (IQR)	1.43 (0.35, 3.84)	2.45 (0.39, 6.12)	0.21	2.80 (0.90, 4.76)	1.40 (0.20, 4.50)	0.21
IgE at onset (U/ml), median (IQR)	1213 (352, 2519)	1109 (453, 2362)	0.98	1085 (325, 2666)	575 (235, 1804)	0.52
ANCA positive at onset, n (%)	20 (33.3)	23 (43.4)	0.33	15 (40.5)	13 (35.1)	0.81
ANCA titer at onset (U/ml), median (IQR) ^b)^	0 (0, 33.8)	0 (0, 73.4)	0.37	0 (0, 84.6)	0 (0, 32.6)	0.41
Initial GC dose (mg/day), median (IQR) ^c)^	50.0 (37.5, 57.5)	50.0 (38.8, 60.0)	0.79	50.0 (40.0, 55.0)	50.0 (30.0, 60.0)	0.72
Initial GC dose (mg/kg), median (IQR) ^c)^	1.0 (0.7, 1.0)	1.0 (0.7, 1.0)	0.91	1.0 (0.8, 1.0)	1.0 (0.6, 1.0)	0.40

Results are expressed as the median (interquartile range) for continuous variables, or the number (%) for nominal variables. Glucocorticoid doses are expressed as prednisolone equivalents. *P<0.05.

^a)^ The patient status at the time of first visit at the three participating institutions. ^b)^ ANCA titers below the detection threshold of 1.0 were recorded as 0. ^c)^ Initial GC oral daily dose during remission induction therapy.

ANCA, antineutrophil cytoplasmic antibody; BVAS, Birmingham vasculitis activity score; CRP, C-reactive protein; FFS, five-factor score; GC, glucocorticoid; IgE, immunoglobulin E; IQR, interquartile range; MPZ, mepolizumab; PSM, propensity score matching.

### 3.2 Patient background after PSM

The PSM identified 37 matched pairs of the MPZ group and the non-MPZ group. After matching, the clinical characteristics, including age at disease onset, sex, disease duration at the last observation, FFS at disease onset, BVAS at disease onset, ANCA positivity at disease onset, and initial GC dose, which is initial GC oral daily dose during remission induction therapy, were comparable between the two groups (**Table 1**). The median age of the 74 patients was 60 years, with 50% being female, and 38% testing ANCA-positive at onset. The frequency of clinical symptoms at onset after matching is reported in **Supplementary Table 1**. The percentage of patients with asthma was 86% (32/37) in the MPZ group and 84% (31/37) in the non-MPZ group, which was lower compared to the MIRRA trial (**Supplementary Table 1**). The median disease duration was 84 months. All patients in the MPZ group after matching received 300 mg of MPZ every 4 weeks. The median duration from MPZ administration to the last observation in the MPZ group was 26 months.

### 3.3 Treatments for EGPA in the MPZ group and non-MPZ group

In the MPZ group, the disease phases consisted of 32 (86.5%) new-onset patients and 5 (13.5%) relapsed patients. The IS used for remission induction therapy were intravenous cyclophosphamide (IVCY) (11 cases) and oral cyclophosphamide (1 case). For remission maintenance therapy before administration of MPZ, azathioprine (AZP) (18 cases), tacrolimus (TAC) (2 cases), rituximab (RTX) (2 cases), methotrexate (MTX) (2 cases), cyclosporine (CyA) (1 case), and MZR (1 case) were administered.

The non-MPZ group consisted of 32 new-onset patients and 5 relapsed patients. The IS used for remission induction therapy included IVCY (11 cases). For remission maintenance therapy, the following medications were administered: AZP (15 cases), RTX (3 cases), TAC (2 cases), CyA (2 cases), and MTX (1 case).

### 3.4 Changes in BVAS and VDI scores

**Figure 1:**
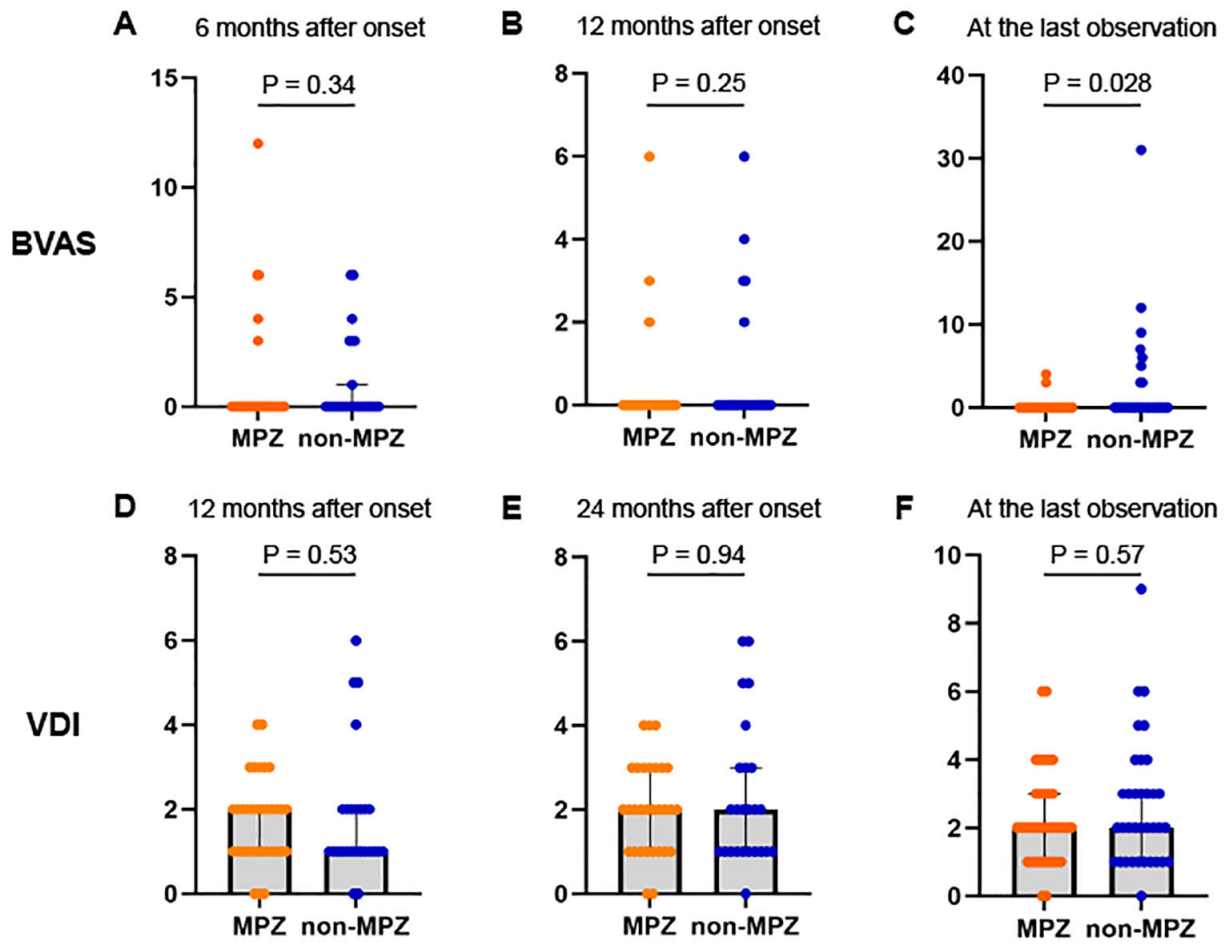
Changes in BVAS and VDI scores between the two groups from six months after onset to the last observation. **(A)** BVAS at 6 months after onset. **(B)** BVAS at 12 months after onset. **(C)** BVAS at the last observation. **(D)** VDI scores 12 months after onset. **(E)** VDI scores 24 months after onset. **(F)** VDI scores at the last observation. Mann-Whitney U test was used to compare differences between the two groups. *P<0.05. BVAS, Birmingham vasculitis activity score; MPZ, mepolizumab; VDI, vasculitis damage index.

We analyzed the longitudinal changes between the two groups using BVAS and VDI. No significant difference in disease duration from onset to the last observation was observed between the two groups (p=0.51, **Table 1**). The serial changes in BVAS in the two groups are shown in **Figures 1A–C**. No significant differences were observed between the two groups in BVAS at 6 and 12 months. The median BVAS at the last observation was 0 in both groups; however, more cases in the non-MPZ group exhibited elevated BVAS, resulting in a significantly higher BVAS in the non-MPZ group at the last observation (median; MPZ group: 0, non-MPZ group: 0, p=0.028). The serial changes in VDI in the two groups at 12 and 24 months and at the last observation are shown in **Figures 1D–F**. There were no significant differences in VDI between the two groups at these time points.

### 3.5 GC-sparing effect of MPZ

**Figure 2:**
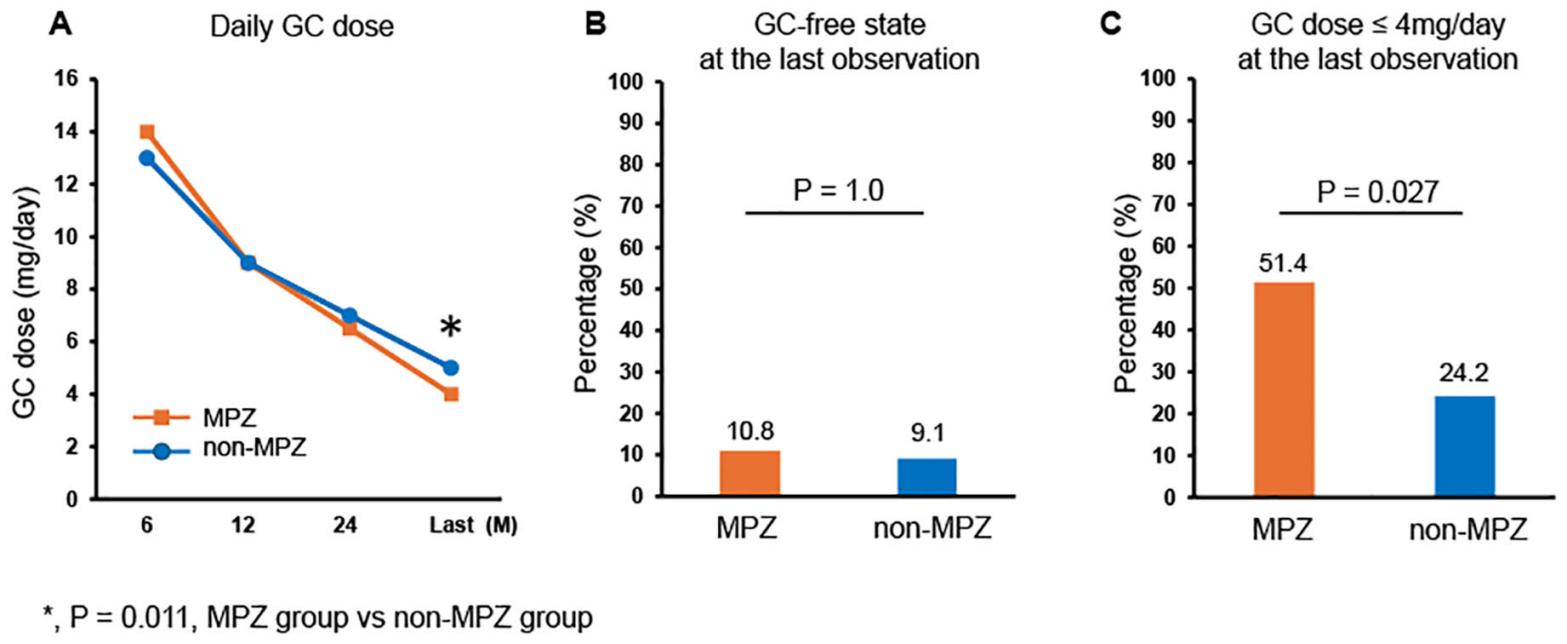
Glucocorticoid-sparing effect of MPZ. **(A)** Serial changes in glucocorticoid dose in the two groups at 6, 12, and 24 months after onset and at the last observation. **(B)** Percentage achieving glucocorticoid-free status at the last observation. **(C)** Percentage achieving glucocorticoid dose ≤ 4 mg/day at the last observation. Fisher’s exact test or the Mann-Whitney U test was used to compare differences between the two groups. *P<0.05. GC, glucocorticoid; MPZ, mepolizumab.

We subsequently evaluated the effect of MPZ on GC tapering by examining the daily GC doses and the percentage achieving GC-free status or GC ≤ 4 mg/day. GC ≤ 4 mg/day was adopted following the remission criteria of the MIRRA trial (11). **Figure 2A** shows the serial changes in the median GC dose (mg/day) between the MPZ group and the non-MPZ group. No differences were observed in the GC dose between the two groups at 6, 12, and 24 months. However, the MPZ group had significantly lower GC doses at the last observation (median: MPZ group: 4 mg/day, non-MPZ group: 5 mg/day, p=0.011). **Figures 2B, C** show a comparison between the two groups in terms of the percentage achieving GC dose-free and GC dose ≤ 4 mg/day at the last observation, respectively. The percentage of patients who achieved a GC dose-free state was similar between the two groups, with 10.8% in the MPZ group and 9.1% in the non-MPZ group (p=1.0). However, the percentage of patients who achieved a GC dose ≤ 4 mg/day was significantly higher in the MPZ group at 51.4%, compared to 24.2% in the non-MPZ group (p=0.027).

### 3.6 Univariable logistic regression analysis of factors involved in achieving GC dose ≤ 4 mg/day

We then sought to identify factors associated with achieving a GC dose ≤ 4 mg/day (**Table 2**). In the univariable analysis, variables included age at onset, gender, disease duration at the last observation, ANCA positivity at onset, FFS at onset, BVAS at onset, eosinophil count at onset, CRP at onset, immunoglobulin E at onset, methylprednisolone pulse at onset, initial GC dose, MPZ administration, and clinical manifestations at onset. MPZ administration (p=0.022) and the presence of asthma in the context of EGPA (p=0.018) were significantly associated with GC dose ≤ 4 mg/day. FFS also showed a trend associated with a GC dose ≤ 4 mg/day (p=0.061).

**Table 2:** Univariable logistic regression analysis of factors associated with achieving a glucocorticoid dose ≤ 4 mg/day.

Variable	OR	95% CI	p
Age at onset	1.00	0.969, 1.04	0.91
Sex	1.13	0.431, 2.96	0.81
Disease duration at the last observation (months)	0.999	0.992, 1.01	0.68
ANCA positivity at onset	1.10	0.403, 2.99	0.86
ANCA titer at onset (U/ml)	0.999	0.997, 1.00	0.54
FFS at onset	1.80	0.973, 3.33	0.061
BVAS at onset	0.992	0.927, 1.06	0.81
White blood cell counts at onset (/μl)	1.00	1.00, 1.00	0.25
Absolute eosinophil count at onset (/μl)	1.00	1.00, 1.00	0.26
CRP at onset (mg/dl)	0.973	0.854, 1.11	0.68
IgE at onset (U/ml)	1.00	1.00, 1.00	0.39
mPSL pulse at onset	0.405	0.141, 1.17	0.094
Initial GC dose, PSL equivalent (mg/day) ^a)^	0.999	0.969, 1.03	0.95
Initial GC dose, PSL equivalent (mg/kg) ^a)^	2.02	0.266, 15.4	0.50
Mepolizumab	3.30	1.18, 9.19	0.022*
Clinical manifestations at onset			
Cutaneous manifestations	2.53	0.928, 6.88	0.070
Mucous membranes/eyes	0.375	0.0397, 3.55	0.39
Asthma	0.149	0.031, 0.718	0.018*
ENT manifestations	1.04	0.372, 2.88	0.95
Cardiovascular involvement	2.22	0.538, 9.12	0.27
Gastrointestinal involvement	5.25	0.517, 53.3	0.16
Renal involvement	0.859	0.254, 2.90	0.81
Peripheral neuropathy	0.982	0.327, 2.95	0.97

*P<0.05. ^a)^ Initial GC oral daily dose during remission induction therapy.

ANCA, antineutrophil cytoplasmic antibody; BVAS, Birmingham vasculitis activity score; CI, confidence interval; CRP, C-reactive protein; ENT, ear, nose, and throat; FFS, five-factor score; GC, glucocorticoid; IgE, immunoglobulin E; mPSL, methylprednisolone; OR, odds ratio; PSL, prednisolone.

### 3.7 Multivariable logistic regression analysis to identify factors achieving GC dose ≤ 4 mg/day

Three models were created for variable selection (**Table 3**). According to the Bonferroni correction, statistical significance was defined as two-sided p values <0.05/3. In Model 1, FFS, MPZ administration, and the presence of asthma in the context of EGPA, which showed significant differences or trends in the univariable analysis, were selected. The results indicated that achieving a GC dose ≤ 4 mg/day was positively associated with MPZ administration (odds ratio [OR] 3.55, 95% CI=1.15–11.0, p=0.028) and was inversely associated with the presence of asthma in the context of EGPA at disease onset (OR 0.103, 95% CI=0.0197–0.544, p=0.0074). In Model 2, MPZ administration, the presence of asthma in the context of EGPA, and ear, nose, and throat (ENT) manifestations, which have been previously reported to be associated with GC dose (8, 11, 25–27), were selected. Similar to Model 1, MPZ administration showed a positive association with achieving a GC dose ≤ 4 mg/day (OR 4.30, 95% CI=1.41–13.1, p=0.010), while the presence of asthma in the context of EGPA had a negative association (OR 0.100, 95% CI=0.0181–0.558, p=0.00865). In Model 3, we added age at onset as a variable in addition to MPZ administration and the presence of asthma in the context of EGPA. This model also indicated that MPZ administration was positively associated (OR 4.29, 95% CI=1.40–13.2, p=0.011) and the presence of asthma in the context of EGPA was negatively associated (OR 0.113, 95% CI=0.0219–0.583, p=0.0092) with achieving GC doses ≤ 4 mg/day.

**Table 3:** Multivariable logistic regression analysis of factors associated with achieving a glucocorticoid dose ≤ 4 mg/day.

Variable	Model 1			Model 2			Model 3		
	OR	95% CI	p	OR	95% CI	p	OR	95% CI	p
Age							0.999	0.962, 1.04	0.96
FFS	1.94	0.927, 4.04	0.079						
Mepolizumab	3.55	1.15, 11.0	0.028*	4.30	1.41, 13.1	0.010*	4.29	1.40, 13.2	0.011*
Asthma	0.103	0.0197, 0.544	0.0074**	0.100	0.0181, 0.558	0.0087**	0.113	0.0219, 0.583	0.0092**
ENT manifestations				1.40	0.4270, 4.580	0.58			

According to the Bonferroni correction, statistical significance was defined as 2-sided p values <0.017. *P<0.05, **P<0.01.

CI, confidence interval; ENT, ear, nose, and throat; FFS, five-factor score; OR, odds ratio.

In addition, a multivariable analysis was conducted on all patients (n=113) before matching, using age at onset, FFS, MPZ administration, the presence of asthma in the context of EGPA, and ENT manifestations as variables. Statistical significance was determined by the Bonferroni correction with a two-sided p value <0.05/5. This analysis also demonstrated a positive association with MPZ administration (OR 3.78, 95% CI=1.53–9.35, p=0.0039) and a negative association with the presence of asthma in the context of EGPA (OR 0.204, 95% CI=0.0641–0.651, p=0.0073) in achieving a GC dose ≤ 4 mg/day (**Supplementary Table 2**).

### 3.8 Asthma treatment

The asthma treatment at the last observation for 17 patients who had asthma symptoms at onset is detailed below. Oral prednisolone (PSL) was administered in all cases, with a median dose of 6 mg/day. Nine patients were receiving inhaled corticosteroids and long-acting β_2_-agonist inhalation therapy, of whom four were also administered long-acting muscarinic antagonist, and two were administered short-acting β_2_-agonist. Mepolizumab, at a dose of 300 mg, was administered to nine patients. Additionally, leukotriene receptor antagonists were prescribed in two cases, and a sustained-release theophylline preparation was used in one case.

### 3.9 The relapse-free survival rate

**Figure 3:**
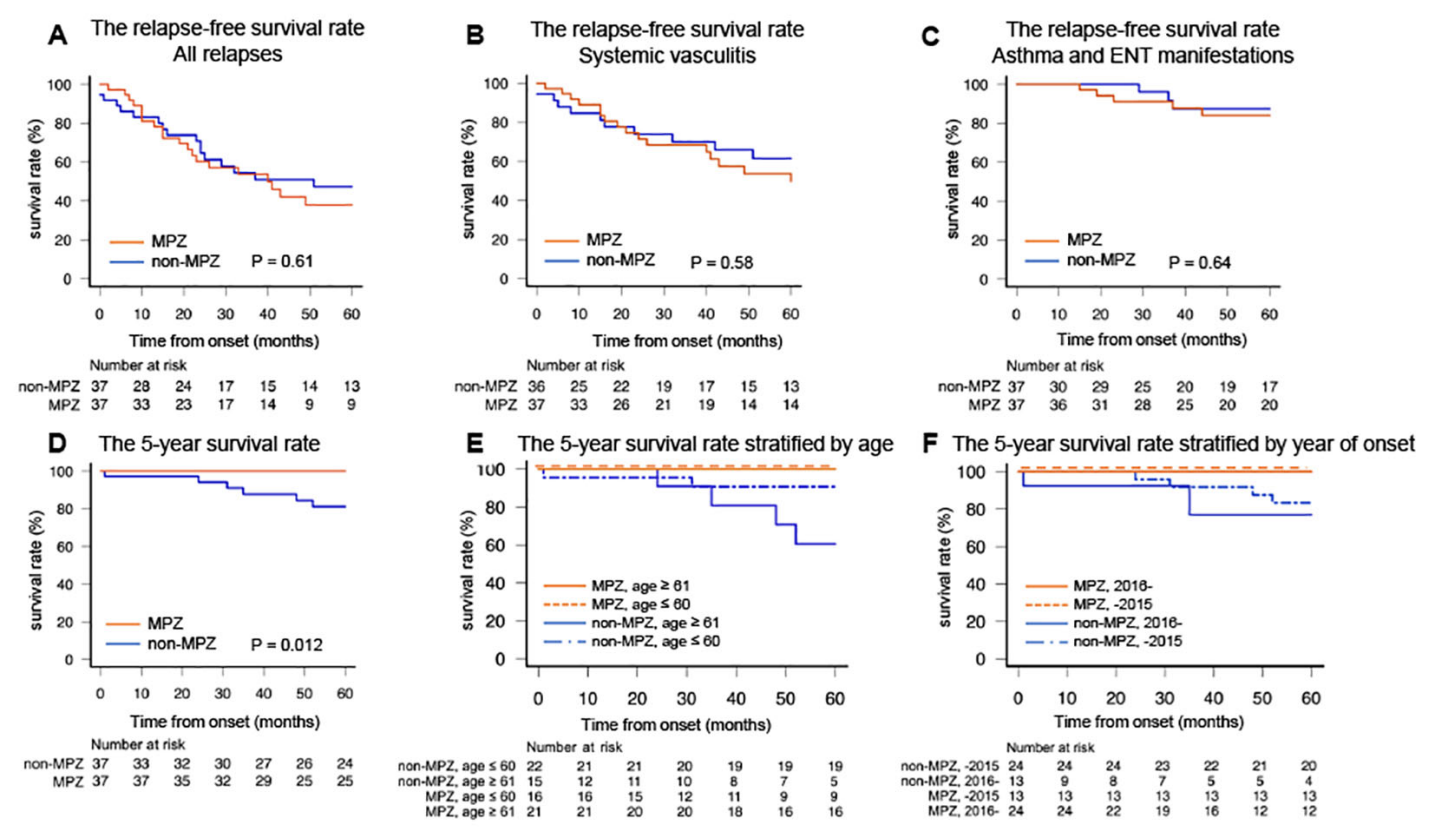
The relapse-free survival rates and five-year survival rates in the two groups. **(A)** The relapse-free survival rate for all relapses. **(B)** The relapse-free survival rate for systemic vasculitis. **(C)** The relapse-free survival rate for asthma and ENT manifestations. **(D)** The five-year survival rate. **(E)** The five-year survival rate stratified by the median age of 60 years. **(F)** The five-year survival rate stratified by the median year of onset in 2015. Relapse-free survival rates and survival rates were calculated by the Kaplan–Meier method and compared using the log-rank test. *P<0.05. ENT, ear, nose, and throat; MPZ, mepolizumab.

In this study, relapse was classified into two categories: relapse due to systemic vasculitis and isolated exacerbation of asthma and ENT manifestations. The five-year relapse-free survival rate for all relapses was not significantly different between the two groups (MPZ group: 38.0%, non-MPZ group: 47.4%, p=0.61, **Figure 3A**). No significant difference in relapse-free survival rate was found between the two groups focusing on relapse of systemic vasculitis (MPZ group: 49.9%, non-MPZ group: 61.5%, p=0.58, **Figure 3B**) as well as exacerbations of asthma and ENT manifestations (MPZ group: 84.1%, non-MPZ group: 87.4%, p=0.64, **Figure 3C**).

Before matching, no significant difference in the relapse-free survival rate, including all relapses, recurrences of systemic vasculitis, and exacerbations of asthma and ENT manifestations, was observed between the MPZ group and the non-MPZ group (**Supplementary Figures 1A-C**).

### 3.10 Comparison of eosinophil counts and ANCA titers between vasculitis relapse and asthma and ENT relapse

The eosinophil count at the time of vasculitis relapse was 470 (99, 947) (median (IQR)), and at the time of asthma and ENT relapse, it was 470 (412, 531), with no significant differences observed (p=1.00). The ANCA titer at the time of vasculitis relapse and at the time of asthma and ENT relapse were 3.6 (0, 22.7) and 0 (0, 0), respectively, with no significant difference noted (p=0.06).

Furthermore, eosinophil counts and ANCA titers between vasculitis and asthma and ENT relapses were compared in the MPZ group and non-MPZ group. In the MPZ group, the eosinophil count at the time of vasculitis relapse was 470 (75, 808), and 420 (256, 460) during asthma and ENT relapses, with no significant difference observed (p=0.70). The ANCA titer at the time of vasculitis relapse was 5.0 (0, 18.9), while it was 0 (0, 0) during asthma and ENT relapses, also with no significant difference (p=0.10). In the non-MPZ group, the eosinophil counts at the time of vasculitis relapse and asthma and ENT relapse were 489 (113, 1361) and 673 (584, 738), respectively, with no significant difference noted (p=0.80). The ANCA titers at the time of vasculitis relapse and asthma and ENT relapse were 0 (0, 214) and 0 (0, 0), respectively, with no significant difference (p=0.68).

### 3.11 The five-year survival rate

Lastly, we examined whether MPZ had an impact on survival. **Figure 3D** shows the five-year survival rates of the MPZ group and the non-MPZ group. The five-year survival rates were significantly higher in the MPZ group compared to the non-MPZ group (MPZ group: 100%, non-MPZ group: 81.3%, p=0.012). To investigate whether the five-year survival rate was influenced by factors such as age or year of onset, we stratified the patients according to the median values for each. When stratified by a median age of 60 years and a median year of onset in 2015, the MPZ group exhibited high survival rates regardless of age or year of onset (**Figures 3E, F**). The five-year survival rates based on median values of FFS at onset, BVAS at onset, eosinophil count at onset, and CRP at onset, which serve as indicators reflecting the severity and activity of EGPA, are shown in **Supplementary Figure 2**. The median values for each indicator in the matched patients were 1 for FFS, 17 for BVAS, 5168 (/μl) for eosinophil count, and 2.1 (mg/dl) for CRP. These values were used to stratify the patients. In all cases, the MPZ group demonstrated high survival rates, independent of disease severity.

Before matching, the five-year survival rate was significantly higher in the MPZ group compared to the non-MPZ group (MPZ group: 100%, non-MPZ group: 87.1%, p=0.011). When stratified by the median age and year of onset, high survival rates were observed in the MPZ group, regardless of age or year of onset (**Supplementary Figures 1D-F**).

## 4 Discussion

In the present study, we evaluated the efficacy of MPZ in patients with EGPA by dividing them into MPZ and non-MPZ groups. Before matching, the MPZ group was significantly younger, which may be due to the fact that MPZ was approved in Japan in 2018. This approval may have led to a tendency to administer MPZ in relatively younger patients who were recently diagnosed with EGPA. Following the matching process, the clinical characteristics of the two groups were found to be comparable.

This study demonstrated that in patients with EGPA, the MPZ group had a lower BVAS at the last observation (**Figure 1C**) and a significantly higher rate of achieving GC ≤ 4 mg/day compared to the non-MPZ group (**Figure 2C**). Multivariable logistic regression analysis demonstrated that achievement of GC ≤ 4 mg/day was positively associated with MPZ administration and negatively associated with wheeze at onset (**Table 3**). Furthermore, the MPZ group exhibited a higher five-year survival rate compared to the non-MPZ group (**Figure 3D**), indicating the potential of MPZ to reduce mortality in EGPA.

We analyzed the serial changes in BVAS and VDI in the two groups. The MIRRA trial defined remission criteria as a BVAS of 0 and a GC dose ≤ 4 mg/day. In the MIRRA trial, patients who were assigned to MPZ administration showed a longer duration of remission and a higher percentage of patients entering remission compared to the placebo group (11). It has been increasingly reported that MPZ controls disease activity and suppresses BVAS in clinical practice as well (28–33). In the current study, IVCY was the most commonly used IS for remission induction therapy, and AZP was used in the majority of cases for remission maintenance therapy. This trend did not significantly differ between the two groups. MPZ was introduced at a median of 32 months after onset, and therefore it is assumed that the BVAS at 6 and 12 months after the onset were not significantly different between the two groups. However, the BVAS in the MPZ group at the last observation was lower compared to the non-MPZ group, even though the required GC dose was significantly reduced. These data suggest that MPZ can effectively control disease activity even when it is introduced several years after diagnosis.

Several recent studies have reported that MPZ suppressed the increase in the VDI score in clinical practice (31, 32, 34, 35). VDI scores have been reported to be higher in older ages, with more frequent recurrences, and with higher GC doses (27, 36). In our study, there were no differences in age or relapse rates between the two groups; however, the GC dose was significantly reduced in the MPZ group compared to the non-MPZ group (**Figure 2A**). Although organ damage caused by long-term GC administration, such as vertebral collapse due to osteoporosis and cataracts, appears to be less common in the MPZ group than in the non-MPZ group (**Supplementary Table 3**) at the last observation, no significant difference was observed in VDI between the two groups. Further accumulation of cases is needed to evaluate whether MPZ can prevent irreversible organ damage.

Regarding VDI, peripheral neuropathy and chronic asthma are particularly common as organ damage, followed by chronic sinusitis, nasal obstruction, and osteoporosis (27, 37). Peripheral neuropathy was the most common in the current study, followed by osteoporosis, chronic asthma, cataracts, and diabetes. The results were consistent with previous reports; however, it should be noted that most of the VDI items, such as osteoporosis, cataracts, and diabetes, are associated with long-term GC use (**Supplementary Table 3**).

The GC-sparing effect of MPZ was demonstrated in the MIRRA trial (11) and other cohort data (28–34, 38). In the current study, the MPZ group received significantly lower GC doses at the last observation, and the percentage of patients achieving GC ≤4 mg/day was significantly higher in the MPZ group, although GC withdrawal was not different between the two groups. It is widely recognized that long-term administration of GC can cause short- and long-term complications, such as hypertension, hyperglycemia, psychiatric conditions, sleep disorders, cataracts, and osteoporosis (39). Increased GC doses carry an increased risk of even fatal complications, including serious infections and cardiovascular events (40, 41). Although there are increasing reports that MPZ administration can successfully lead to GC withdrawal in 40-50% of patients (28, 33), our study results indicate that achieving complete GC withdrawal remains a challenge.

The results of the multivariable analysis suggest that patients with EGPA complicated by asthma have difficulty in reducing the GC dose in the current study (**Table 3**, **Supplementary Table 2**). Given that the median dose of PSL at the last observation for all matched patients was 5 mg, the fact that it was 6 mg in patients who had asthma symptoms at onset further supports this finding. In the management of asthma, as-needed low-dose oral corticosteroids (OCS) should be considered (42); however, high-dose OCS remain widely used for patients with uncontrolled asthma (43, 44). Given the side effects of OCS, it is essential to optimize topical therapies, such as inhaled corticosteroids and bronchodilators, as well as systemic medications including azithromycin and leukotriene receptor antagonists (8, 42). MPZ is also effective in treating asthma, and its proactive administration should be considered for patients with EGPA who present with concomitant asthma (8, 29, 44). The appropriate management of asthma symptoms requires close collaboration with pulmonologists (8).

Asthma is present in almost all patients with EGPA (8). However, in the current study, 14% of the MPZ group and 16% of the non-MPZ group were not diagnosed with asthma (**Supplementary Table 1**). Asthma symptoms fluctuate over time, with variations in intensity and the development of airflow limitation, making diagnosis challenging (42). Additionally, asthma patients, particularly younger individuals, often have poor awareness of their symptoms (45). Moreover, EGPA patients are frequently first assessed by rheumatologists rather than pulmonologists or allergists. These factors suggest that even among patients not diagnosed with asthma in our study, some patients may have had mild asthma. In such patients, tapering GC may not be particularly challenging compared to EGPA patients identified as having coexisting asthma.

Subsequently, we evaluated the relapse-free survival rate as a long-term outcome. In the MIRRA trial, the MPZ group had a lower percentage of patients experiencing relapse compared to the placebo group (11). However, in this study, no differences were observed between the two groups in terms of all relapses, relapses of systemic vasculitis, and relapses of asthma and ENT manifestations (**Figures 3A–C**). In addition to the delay in the introduction of MPZ, ANCA status may be another reason for the failure of the MPZ group to prevent relapses in this study. The relationship between ANCA status and the risk of relapse is controversial (8); however, several studies have shown that the five-year relapse rate was high in ANCA-positive cases (35–46%) compared with that in ANCA-negative cases (22–35%) (26, 46). In the current study, the ANCA-positive rate was 38%, which is higher than the 19% reported in the MIRRA study (**Supplementary Table 1**). When the BVAS at the last observation was categorized according to ANCA status, the significantly higher scores in the ANCA-positive group would further support this hypothesis (**Supplementary Figure 3A**). The higher rate of MPO-ANCA positivity may have made it more challenging to suppress relapses with MPZ.

Regarding relapse symptoms, in the MIRRA trial, 20% were vasculitic relapses, and 54% were relapses of asthma or sinusitis (11). In the current study, the five-year relapse rates of systemic vasculitis and asthma and ENT manifestations were 44.9% and 14.3%, respectively, indicating a higher relapse rate for vasculitis and a lower relapse rate for asthma and ENT manifestations. Three reasons may have contributed to this difference. One consideration is that the ANCA positivity rate in this study was higher than in the MIRRA trial. The pathophysiology of EGPA is becoming more widely recognized as comprising two distinct mechanisms: T2-eosinophilic inflammation, such as asthma and ENT manifestations, and ANCA-positive vasculitis, such as nephritis and purpura (3, 5, 47). Based on this dualism, the high ANCA positivity rate observed in the current study could have contributed to the higher relapse rate of vasculitis.

Furthermore, in recent years, several cases have been reported where patients undergoing anti-IL-5 therapy targeting T2-eosinophilic inflammation experienced a relapse of vasculitis in EGPA, marked by an increase in ANCA levels, despite having normal eosinophil counts (48, 49). These reports suggest that ANCA plays an exclusive role in the vasculitic pathophysiology of EGPA. In our cohort, two patients developed hemorrhagic infarction and myocarditis that were judged by the attending physician to be relapse of vasculitis, presenting with high ANCA levels despite low eosinophil counts following methylprednisolone pulse and MPZ. However, after matching, no significant differences were observed in eosinophil counts or ANCA titers between vasculitis relapse and asthma and ENT relapse. The roles of eosinophils and ANCA in the pathophysiology of EGPA are still under debate. Therefore, it is crucial to further promote the accumulation and analysis of cases of vasculitis relapse in patients undergoing anti-IL-5 therapy.

Another potential factor contributing to the high frequency of vasculitis relapse observed in our study is that, while the prevalence of ENT and asthma at onset was 94% and 100%, respectively, in the MIRRA trial, this study reported lower rates of 34% and 85% (**Supplementary Table 1**). However, with regard to the diagnosis of asthma, as previously mentioned, there is a possibility that mild asthma symptoms may have been overlooked in this cohort, requiring caution in this assessment. The final reason may be the difference in the use of IS compared to the MIRRA trial. Specifically, in the MIRRA trial, 77.2% (105/136) of patients received IS after diagnosis of EGPA, whereas in our study, the rate was lower at 63.5% (47/74). This difference may have contributed to the higher relapse rate of ANCA-mediated inflammation.

In addition, the five-year survival rates were evaluated. The five-year survival rate for systemic vasculitis was 72.2% prior to 1980, but it has improved over time, and the recent five-year survival rate for EGPA is relatively high at approximately 88–97% (26, 50, 51). Before 2000, the primary causes of death in ANCA-associated vasculitis were exacerbations of vasculitis and infections due to treatment. However, with improvements in the treatment of vasculitis, malignancies have increased since 2010 (50). Cardiac involvement also remains the primary cause of death in EGPA, accounting for one-third of all mortalities (47, 52). In the current study, eight out of 113 patients before matching died during the observation period. Among these, six patients died within five years of onset, resulting in a five-year survival rate of 94.7%. The causes of death were exacerbation of EGPA in 3 patients, infection in 2 patients, and malignancy in 2 patients (**Supplementary Table 4**).

The mortality rate of EGPA may differ depending on ANCA status. In particular, an increased risk of mortality in ANCA-negative cases has been reported, which may be attributable to a higher prevalence of cardiac involvement in ANCA-negative patients (46, 53). Out of the eight patients who died in this study, only two were MPO-ANCA positive, and the negative cases included a patient who died due to cardiac involvement (**Supplementary Table 4**). However, among the 113 patients before matching, the survival rates were equivalent between ANCA-positive and ANCA-negative patients (ANCA-positive: 94.9%, ANCA-negative: 92.9%, p=0.82). Furthermore, we compared the VDI and GC doses at the last observation based on ANCA status. However, no significant differences were observed in either comparison (**Supplementary Figures 3B, C**). Several reports suggest that there is no difference in mortality rates based on ANCA status, indicating that a consensus has not yet been reached (8, 27).

Importantly, a point worthy of mentioning in our study is the potential impact of MPZ on the long-term prognosis of EGPA. The MPZ group showed high survival rates regardless of age, year of onset, or disease severity. Taken together with the above-mentioned findings, it is speculated that MPZ controlled the disease activity of EGPA and reduced the GC dose, ultimately leading to the high survival rate.

This study has several limitations. The first concern is that the current study is a retrospective study with a small number of cases. The second limitation concerns remaining background biases, which were not completely excluded by PSM. Particularly, MPZ might have been administered to patients who had already survived a long period of morbidity, indicating that survival bias may not have been completely eliminated. The third limitation is that variations in clinical treatment strategies and patient characteristics among different hospitals could potentially act as confounding factors or biases. Despite these limitations, our study results, for the first time, demonstrated that MPZ may improve the long-term prognosis in patients with EGPA. Further case accumulation is necessary to validate our study results.

## 5 Conclusion

MPZ may control disease activity and reduce the required GC doses, potentially improving the long-term prognosis in patients with EGPA.

## Data Availability

The raw data supporting the conclusions of this article will be made available by the authors, without undue reservation.
