# Supplementary material for: Long-term efficacy of mepolizumab in patients with eosinophilic granulomatosis with polyangiitis: a propensity score matching analysis in the multicenter REVEAL cohort study
Source: Front Immunol. 2024 Oct 2;15:1457202. doi: 10.3389/fimmu.2024.1457202 (PMC11479934; doi:10.3389/fimmu.2024.1457202)

Supplementary Material

# Supplementary Table

**Supplementary Table 1. Comparison of baseline characteristics between the MIRRA study and this study.**

|  | MIRRA trial | |  | This study (after matching) | |
| --- | --- | --- | --- | --- | --- |
| Variable | MPZ  (n=68) | Placebo  (n=68) |  | MPZ  (n=37) | non-MPZ  (n=37) |
| Female sex, n (%) | 42 (62) | 38 (56) |  | 21 (57) | 16 (43) |
| ANCA-positive status, n (%) | 13 (19) | 13 (19) |  | 15 (41) | 13 (35) |
| Duration since diagnosis (years) | 5.2 | 5.9 |  | 6.0 | 7.7 |
| Biopsy evidence, n (%) | 25 (37) | 31 (46) |  | 20 (54) | 17 (46) |
| Asthma with eosinophilia, n (%) | 68 (100) | 68 (100) |  | 32 (86) | 31 (84) |
| Neuropathy, n (%) | 32 (47) | 24 (35) |  | 31 (84) | 29 (78) |
| Nonfixed pulmonary infiltrates, n (%) | 50 (74) | 48 (71) |  | 14 (38) | 17 (46) |
| Sinonasal abnormality, n (%) | 64 (94) | 64 (94) |  | 23 (62) | 15 (41) |
| Cardiomyopathy, n (%) | 13 (19) | 7 (10) |  | 6 (16) | 4 (11) |
| Glomerulonephritis, n (%) | 1 (1) | 0 |  | 8 (22) | 8 (22) |
| Alveolar hemorrhage, n (%) | 3 (4) | 1 (1) |  | 2 (5) | 1 (3) |
| Palpable purpura, n (%) | 9 (13) | 8 (12) |  | 19 (51) | 14 (38) |

Results are expressed as the number (%) for nominal variables.

ANCA, antineutrophil cytoplasmic antibody; MPZ, mepolizumab.

**Supplementary Table 2. Multivariable logistic regression analysis of factors associated with achieving a glucocorticoid dose ≤ 4mg/day before matching.**

|  | Univariable | | |  | Multivariable | | |
| --- | --- | --- | --- | --- | --- | --- | --- |
| Variable | OR | 95% CI | p |  | OR | 95% CI | p |
| Age | 0.985 | 0.961, 1.01 | 0.23 |  | 0.972 | 0.940, 1.00 | 0.096 |
| Sex | 1.060 | 0.490, 2.29 | 0.88 |  |  |  |  |
| Disease duration (months) | 0.998 | 0.993, 1.00 | 0.50 |  |  |  |  |
| ANCA positivity | 0.728 | 0.324, 1.63 | 0.44 |  |  |  |  |
| ANCA titer (U/ml) | 0.998 | 0.996, 1.00 | 0.24 |  |  |  |  |
| FFS | 1.26 | 0.803, 1.980 | 0.32 |  | 1.93 | 1.02, 3.65 | 0.044* |
| BVAS | 0.986 | 0.939, 1.04 | 0.58 |  |  |  |  |
| White blood cell counts (/μl) | 1.00 | 1.00, 1.00 | 0.24 |  |  |  |  |
| Absolute eosinophil count (/μl) | 1.00 | 1.00, 1.00 | 0.33 |  |  |  |  |
| CRP (mg/dl) | 0.949 | 0.853, 1.06 | 0.33 |  |  |  |  |
| IgE (U/ml) | 1.00 | 1.00, 1.00 | 0.11 |  |  |  |  |
| mPSL pulse | 0.452 | 0.201, 1.01 | 0.054 |  |  |  |  |
| Initial GC dose, PSL equivalent (mg/day) | 0.995 | 0.971, 1.02 | 0.72 |  |  |  |  |
| Initial GC dose, PSL equivalent (mg/kg) | 1.010 | 0.205, 4.99 | 0.99 |  |  |  |  |
| Mepolizumab | 3.43 | 1.50, 7.84 | 0.0035* |  | 3.78 | 1.53, 9.35 | 0.0039** |
| Clinical manifestations at onset |  |  |  |  |  |  |  |
| Cutaneous manifestations | 1.74 | 0.793, 3.80 | 0.17 |  |  |  |  |
| Mucous membranes/eyes | 0.566 | 0.105, 3.06 | 0.51 |  |  |  |  |
| Asthma | 0.349 | 0.127, 0.960 | 0.041 |  | 0.204 | 0.0641, 0.651 | 0.0073** |
| ENT manifestations | 1.100 | 0.493, 2.47 | 0.81 |  | 1.890 | 0.730, 4.89 | 0.19 |
| Cardiovascular involvement | 1.570 | 0.540, 4.57 | 0.41 |  |  |  |  |
| Gastrointestinal involvement | 2.29 | 0.366, 14.3 | 0.38 |  |  |  |  |
| Renal involvement | 0.800 | 0.303, 2.11 | 0.65 |  |  |  |  |
| Peripheral neuropathy | 0.909 | 0.371, 2.23 | 0.84 |  |  |  |  |

According to the Bonferroni correction, statistical significance was defined as 2-sided p values <0.01. *P<0.05, **P<0.01

ANCA, antineutrophil cytoplasmic antibody; BVAS, Birmingham vasculitis activity score; CI, confidence interval; CRP, C-reactive protein; ENT, ear, nose, and throat; FFS, five-factor score; GC, glucocorticoid; IgE, immunoglobulin E; mPSL, methylprednisolone; OR, odds ratio; PSL, prednisolone.

**Supplementary Table 3**. **Common organ failures of VDI in our study after matching.**

| Variable | MPZ (n=37) | non-MPZ (n=35) | p |
| --- | --- | --- | --- |
| Peripheral neuropathy, n (%) | 22 (59.5) | 16 (45.7) | 0.35 |
| Osteoporosis vertebral collapse, n (%) | 10 (27) | 12 (34.3) | 0.61 |
| Chronic asthma, n (%) | 13 (35.1) | 6 (17.1) | 0.11 |
| Cataract, n (%) | 4 (10.8) | 9 (25.7) | 0.13 |
| Diabetes, n (%) | 7 (18.9) | 4 (11.4) | 0.52 |
| Malignancy, n (%) | 3 (8.1) | 5 (14.3) | 0.47 |
| Chronic sinusitis/radiological damage, n (%) | 5 (13.5) | 2 (5.7) | 0.43 |
| Complicated venous thrombosis, n (%) | 2 (5.4) | 3 (8.6) | 0.67 |
| Estimated / measured GFR ≤ 50%, n (%) | 1 (2.7) | 4 (11.4) | 0.19 |
| Avascular necrosis, n (%) | 1 (2.7) | 3 (8.6) | 0.35 |
| Impaired lung function, n (%) | 3 (8.1) | 1 (2.9) | 0.62 |
| Cerebrovascular accident, n (%) | 2 (5.4) | 2 (5.7) | 1.0 |

The non-MPZ group has n=35 due to missing data. Results are expressed as the number (%) for nominal variables. Differences between the two groups were compared using Fisher's exact test. *P<0.05.

GFR, glomerular filtration rate; MPZ, mepolizumab; VDI, vasculitis damage index.

**Supplementary Table 4. Cause of death in 8 cases before matching.**

|  | Age at onset | Age at the death | Months from onset to death | Sex | MPO-ANCA | Cause of death |
| --- | --- | --- | --- | --- | --- | --- |
| Exacerbation of EGPA | 59 | 59 | 1 | female | positive | subcortical hemorrhage |
|  | 38 | 59 | 252 | male | negative | sustained ventricular tachycardia |
|  | 36 | 38 | 31 | female | negative | macrophage activation syndrome |
| Infection | 79 | 82 | 35 | male | positive | aspiration pneumonia |
|  | 70 | 72 | 24 | female | negative | septic shock |
| Malignancy | 65 | 69 | 48 | female | negative | DLBCL |
|  | 83 | 94 | 132 | female | negative | bladder cancer |
|  | 75 | 80 | 52 | female | negative | breast cancer |

ANCA, antineutrophil cytoplasmic antibody; DLBCL, diffuse large B-cell lymphoma; EGPA, eosinophilic granulomatosis with polyangiitis; MPO, myeloperoxidase.

# Supplementary Figures

**Supplementary Figure 1. The relapse-free survival rates and five-year survival rates before matching.** (A) The relapse-free survival rate for all relapses. (B) The relapse-free survival rate for systemic vasculitis. (C) The relapse-free survival rate for asthma and ENT manifestations. (D) The five-year survival rate. (E) The five-year survival rate stratified by the median age of 59 years. (F) The five-year survival rate stratified by the median year of onset in 2016. Relapse-free survival rates and survival rates were calculated by the Kaplan–Meier method and compared using the log-rank test. *P<0.05. ENT, ear, nose, and throat; MPZ, mepolizumab. **
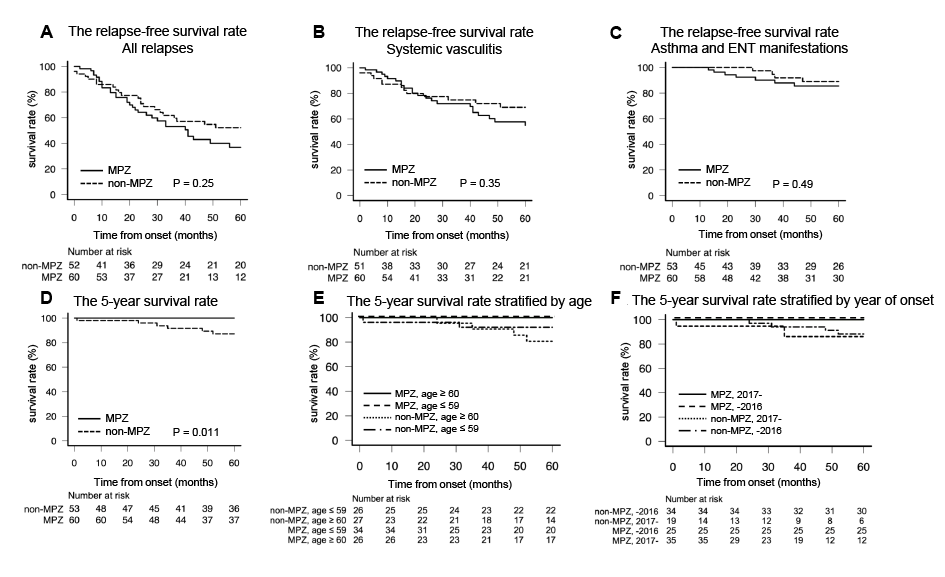
**

**Supplementary Figure 2.** **The five-year survival rates after matching stratified by indicators of severity at disease onset.** (A) The five-year survival rate stratified by the median FFS of 1 at onset. (B) The five-year survival rate stratified by the median BVAS of 17 at onset. (C) The five-year survival rate stratified by the median eosinophil count of 5168 /µL at onset. (D) The five-year survival rate stratified by the median CRP of 2.1 mg/dL at onset. BVAS, Birmingham vasculitis activity score; CRP, C-reactive protein; FFS, five-factor score; MPZ, mepolizumab.

**
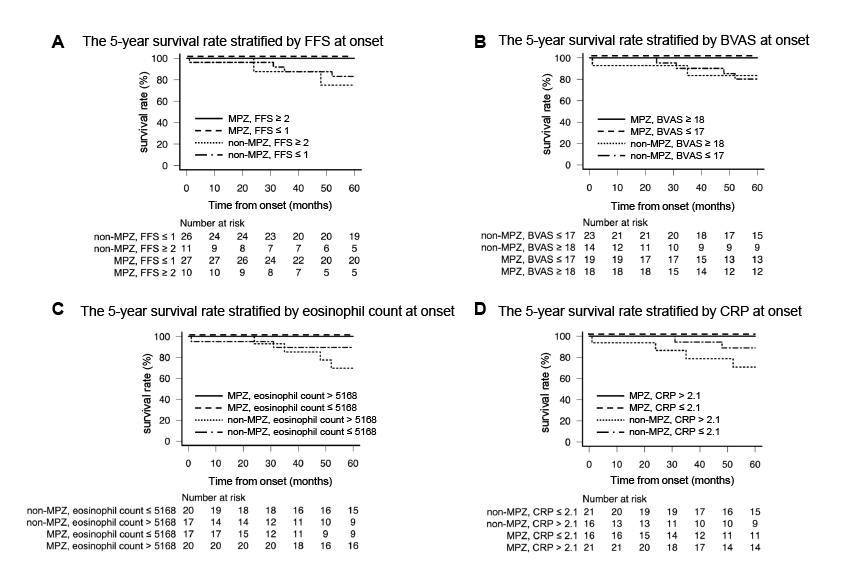
**

**Supplementary Figure 3. BVAS, VDI score, and GC dose at the last observation with a focus on ANCA status after matching.** (A) BVAS at the last observation stratified by ANCA status. (B) VDI at the last observation stratified by ANCA status. (C) GC dose at the last observation stratified by ANCA status. ANCA, antineutrophil cytoplasmic antibody; BVAS, Birmingham vasculitis activity score; GC, glucocorticoid; VDI, vasculitis damage index.


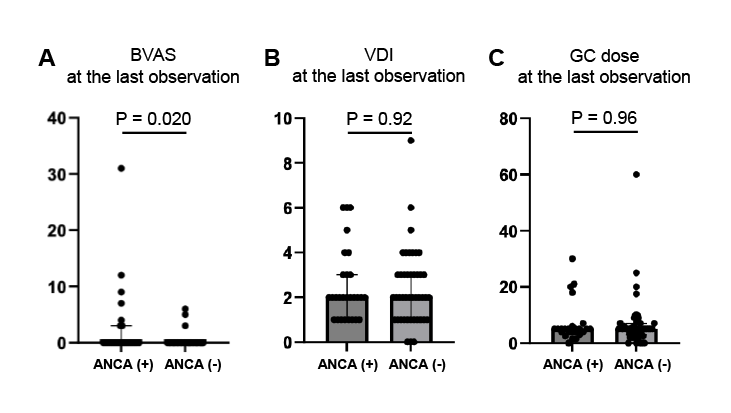

Supplement: Supplementary file 1 [file DataSheet1.docx]
